# Differential regulation of mRNAs and lncRNAs related to lipid metabolism in two pig breeds

**DOI:** 10.18632/oncotarget.20978

**Published:** 2017-09-18

**Authors:** Wanlong Huang, Xiuxiu Zhang, Ai Li, Lingli Xie, Xiangyang Miao

**Affiliations:** ^1^ State Key Laboratory of Animal Nutrition, Institute of Animal Sciences, Chinese Academy of Agricultural Sciences, Beijing 100193, China

**Keywords:** lncRNA, subcutaneous adipose tissue, fat deposition, pig, RNA-seq

## Abstract

Long non-coding RNAs (lncRNAs) can regulate lipid metabolism and adipogenesis. However, there is little research on the role of lncRNAs in fat deposition in pig. In this study, RNA-seq technology was used to analyze the gene expression profiles of subcutaneous adipose tissue in Laiwu (LW) and Large White (LY) pigs. Then, key lncRNAs and genes associated with lipid metabolism and adipogenic differentiation were identified. Fifty four lncRNAs and 482 known mRNAs were differentially expressed in the two pig breeds. GO (Gene Ontology) and KEGG (Kyoto Encyclopedia of Genes and Genomes) pathway analyses revealed that differentially expressed genes and the target genes of differentially expressed lncRNAs were significantly enriched in PPAR signaling pathway and biological processes including fat cell differentiation and fatty acid metabolism. Key lncRNAs might regulate adipogenic differentiation and fatty acid metabolism by regulating genes involved in above signaling pathway and biological processes. Specifically, *XLOC_014379*, *XLOC_011279*, *XLOC_064871*, *XLOC_019518* and *XLOC_013639* might target *SCD*, *LPIN1*, *TRIB3*, *EGR2* and *FABP3*, respectively, and then play critical regulatory role. These results are useful for understanding fat deposition in pig, breeding livestock with high quality meat, and preventing and treating lipid metabolic disease.

## INTRODUCTION

Domestic pigs (Sus scrofa domesticus) are the major species for meat consumption worldwide. Fat deposition in pigs varies with breeds. Backfat thickness has a strong influence on fattening efficiency, reproductive performance, growth rate and intramuscular fat content of pig. However, breeding pig with high proportion of lean meat in the long term have led to lower intramuscular fat content and finally decreased pork quality [1]. Therefore, how to breed pig species with low backfat thickness that can produce high quality meat remains a challenge. Meanwhile, excessive fat deposition in human body can result in obesity and disorders of energy metabolism, which further leads to obesity-associated diseases, such as type II diabetes, insulin resistance, cardiovascular diseases and some cancer [2]. Thus, human health problems caused by obesity are attracting more attention. Importantly, pig is anatomically and physiologically similar to human, so it can be a good medical model for studying lipid metabolic disease. Better clarification of the molecular basis of fat deposition in pigs will deepen our understanding of diseases associated with fat metabolism. Previous research has shown that adipogenic differentiation and lipid metabolism are regulated by transcription factors and signaling pathways [3, 4]. The transcription factors include bone morphogenetic protein 4 (BMP4), peroxisome proliferator activated receptor gamma (PPARγ), CCAAT/enhancer binding proteins (C/EBPs), adipocyte determination and differentiation dependent factor 1 (ADD1), Kruppel-like factors (KLFs) and zinc finger protein 423 (ZFP423). The signaling pathways include PPAR, mitogen-activated protein kinase (MAPK), transforming growth factor β (TGF β) and Insulin. Several genes have been identified to regulate adipogenesis and lipid metabolism in pig, including *KLF13* [5], fibroblast growth factor (*FGF21*) [6], lipin 1 (*LPIN1*) [7] and stearoyl-CoA desaturase (*SCD*) [8]. However, the regulatory mechanisms have not yet been elucidated completely.

LncRNAs, non-protein coding transcripts longer than 200 nucleotides, have important regulatory functions. RNA-seq has high sensitivity in identifying differentially expressed genes as well as comprehensively characterizing and quantitatively analyzing transcriptome [9, 10]. It has been widely applied in the identification and functional analysis of lncRNAs and mRNAs of adipose tissues in pig [8, 11], chicken [12], sheep [13, 14] and cattle [15]. Ramayo et al [16] identified 270 and 186 lncRNAs in the liver tissues of female pigs with high and low intramuscular fatty acid contents, respectively. Zhou et al [11] analyzed the methylation of lncRNA and found the expression level of linc-sscg3623 varied with pig breeds and developmental stages, affecting fat synthesis. Zhang et al [12] analyzed the lncRNA and mRNA expression profiles of preadipocytes at different stages in abdominal adipose tissue of Jinghai Yellow chicken. They found that the target genes of lncRNAs were significantly enriched in MAPK and PPAR signaling pathway associated with adipocyte differentiation. These studies indicate that lncRNAs might determine fat deposition and fatty acid composition, and regulate adipogenic differentiation and lipid metabolism in livestock and poultry. However, there is limited research on the expression profiles and the functions of lncRNAs in subcutaneous adipose tissue of pig breeds with significant differences in fat deposition.

Laiwu (LW) pig, an excellent fatty breed of North China, is characterized by high quality meat with bright color, high water-holding capacity and especially high intramuscular fat content (10.32%) [17]. Compared with Erhualian, Laiwu and Lulai Black pigs [18, 19], Large White (LY) pig, the most widely distributed lean-type pig breed, has lower contents of subcutaneous and intramuscular fat. Thus, LW and LY pigs provide good resources for studying fat deposition, adipogenic differentiation and diseases associated with fat metabolism.

In this study, RNA-seq technology was used to comparatively analyze the gene expression profiles of subcutaneous adipose tissue between LW and LY pigs. Then, key lncRNAs and genes associated with adipogenesis and lipid metabolism were identified. Furthermore, with the help of Gene Ontology (GO), Kyoto Encyclopedia of Genes and Genomes (KEGG) pathway, Co-expression and protein-protein interaction (PPI) network analyses, we investigated the molecular mechanism of differentially expressed lncRNAs and genes regulating fat deposition. The results can provide useful information for studying fat deposition regulated by lncRNAs in porcine adipose tissue. In addition, this study is useful for studying lipid metabolic disease and breeding livestock and poultry with high quality meat.

## RESULTS

### Total RNA sequencing and mapping

To comprehensively understand the transcriptomes of subcutaneous fat tissues in LW and LY pigs, total RNA was isolated and sequenced by Illumina sequencing platform. Approximately 11 Gb sequencing data per sample were obtained. After pre-processing and low-quality trimming of the sequencing data, about 90 million clean reads were obtained for each sample. About 71.27–76.90% clean reads were mapped to the reference genome of pig and 60% were uniquely mapped (Table 1). By assembly and reconstruction, a total of 92,508 transcripts (FPKM ≥ 0.01) were obtained. Among them, 21,644 were protein-coding transcripts.

**Table 1:** Summary of raw reads after quality control and mapping to the reference genome

Sample	Raw reads	Clean reads	Valid ratio (%)	Q30 (%)	GC content (%)	Total mappedreads	Uniquely mappedreads
LY1	94810516	90927088	95.84%	94.61%	51.50%	69921634 (76.9%)	57700161 (63.46%)
LY2	92647174	88999976	96.00%	94.73%	51.50%	68031369 (76.44%)	56194896 (63.14%)
LY3	95069640	91532758	96.22%	94.78%	55.00%	68970804 (75.35%)	54923704 (60%)
LW1	92860394	89189416	95.98%	95.28%	51.00%	66219222 (74.25%)	54266624 (60.84%)
LW2	94815260	91416676	96.34%	95.43%	52.00%	65157231 (71.27%)	55934087 (61.19%)
LW3	92647706	88944438	95.94%	95.18%	50.00%	65611080 (73.77%)	54756763 (61.56%)

### Identification and characterization of lncRNAs

**Figure 1:**
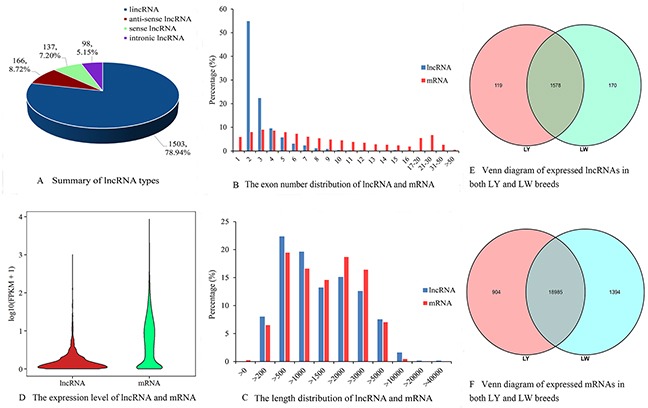
LncRNA characterization and gene expression **(A)** Summary of lncRNA types. **(B)** The exon number distribution of lncRNA and mRNA. **(C)** The length distribution of lncRNA and mRNA. **(D)** The expression level of lncRNA and mRNA. **(E)** Venn diagram of expressed lncRNAs in both LY and LW breeds. **(F)** Venn diagram of expressed mRNAs in both LY and LW breeds.

A total of 1904 lncRNA candidates were identified, including 1503 lincRNAs, 166 anti-sense lncRNAs, 137 sense lncRNAs and 98 intronic lncRNAs in the six libraries (Figure 1A). LincRNA was the most common type of lncRNAs and its proportion was also the largest, which were consistent with current research. Among all the lncRNAs, 771 (about 40.49%) were mapped to ALDB database. As shown in Figure 1B, most lncRNAs had two exons (an average of 3.01 exons), which were significantly less than the exons of protein-coding gene (up to 118 exons, an average of 9.62 exons). In addition, the amount of exons in lncRNAs in subcutaneous adipose tissue of pig was consistent with that in thyroid [20] and testicular tissue [21] of pig. Overall, the distributions of lncRNA and protein-coding gene lengths were consistent, and the proportion of relatively long mRNA transcripts was higher than that of lncRNAs (Figure 1C). The average length of lncRNAs and mRNAs was 2314 and 2225 nt, respectively.

### Expression level of genes and differential expression analysis

Violin plot of FPKM values for the transcripts indicated that the expression level of mRNAs was relatively higher than that of lncRNAs (Figure 1D), which was consistent with the research on lncRNAs in pig testicular tissue [21], mouse [22] and human [23]. FPKM values for most lncRNAs were less than 10, whereas those for mRNAs were evenly distributed. A total of 119 lncRNAs and 904 mRNAs were expressed specifically in subcutaneous adipose tissues of LY pig, and 170 lncRNAs and 1394 mRNAs were expressed specifically in subcutaneous adipose tissues of LW pig (Figure 1E and 1F). Differential expression analysis ((LW *vs* LY) indicated that 40 lncRNAs were up-regulated and 14 were down-regulated in LW pig (Supplementary Table 1). A total of 482 known differentially expressed mRNAs were identified. Of these, 223 mRNAs were up-regulated in LW pig and the rest were down-regulated (Supplementary Table 2).

### GO annotation of differentially expressed genes

**Figure 2:**
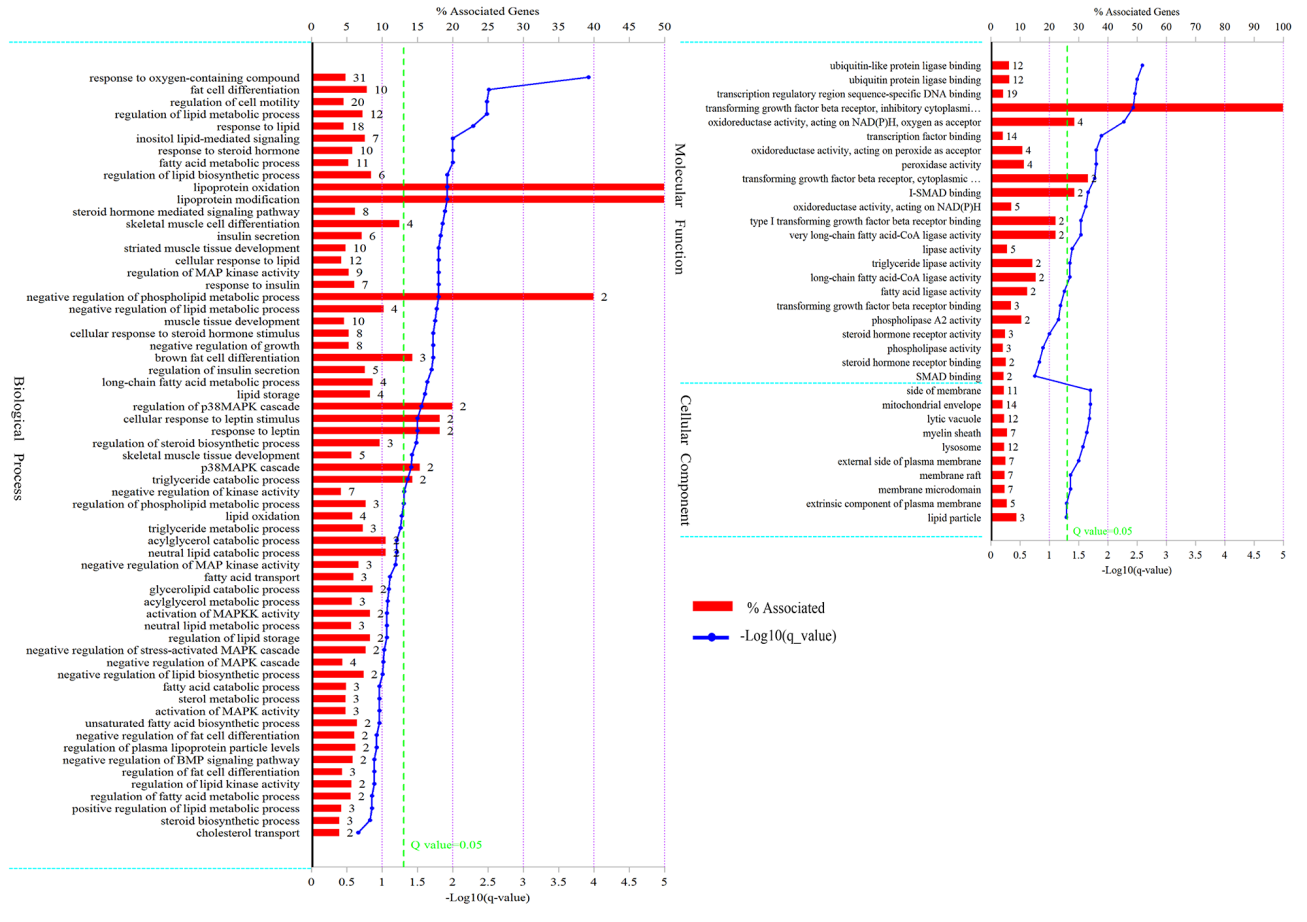
GO analysis of differentially expressed genes The figure is composed of three parts: biological processes, molecular functions, and cellular components. The significance level of enrichment was set at corrected P value (Q value) < 0.05.

A total of 211 differentially expressed genes were annotated in GO terms (Supplementary Table 3). As shown in Figure 2, in the category of biological process, differentially expressed genes were mainly enriched in “response to oxygen-containing compound”, “fat cell differentiation”, “regulation of lipid metabolic process”, “fatty acid metabolic process”, “response to steroid hormone”, etc. In the category of molecular function, differentially expressed genes were mainly enriched in “ubiquitin-like protein ligase binding”, “transcription regulatory region sequence-specific DNA binding”, “lipase activity”, etc. In the category of cellular component, differentially expressed genes were mainly enriched in “mitochondrial envelope”, “side of membrane”, etc. The differentially expressed genes in LY and LW pigs should mainly regulate adipocyte differentiation and lipid metabolism.

### KEGG enrichment analysis of differentially expressed genes

**Figure 3:**
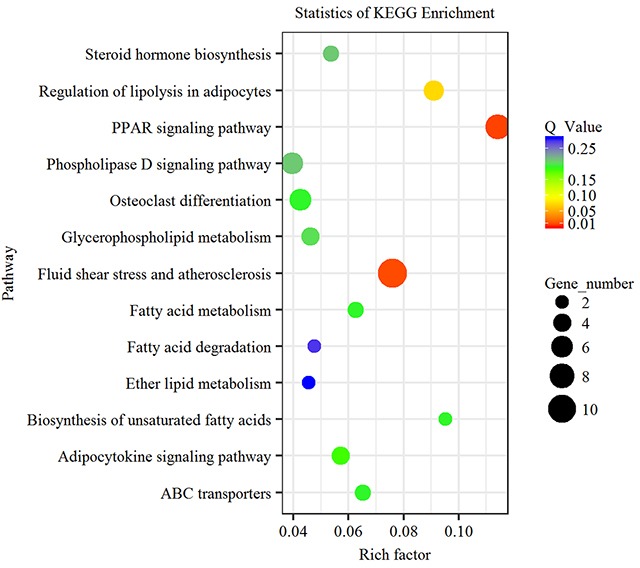
KEGG pathway analysis of differentially expressed genes Advanced bubble chart shows enrichment of differentially expressed genes in signaling pathways. Y-axis label represents pathway, and X-axis label represents rich factor (rich factor = amount of differentially expressed genes enriched in the pathway/amount of all genes in background gene set). Size and color of the bubble represent amount of differentially expressed genes enriched in the pathway and enrichment significance, respectively.

KEGG enrichment analysis demonstrated that 115 differentially expressed genes were enriched in 63 signaling pathways. Among these signaling pathways, five were significantly enriched (Supplementary Table 4). PPAR signaling pathway (Q value=0.0097) and fluid shear stress and atherosclerosis (Q value=0.012) were the two most significantly enriched signaling pathways. In addition, differentially expressed genes were also enriched in signaling pathways associated with lipid metabolism, such as regulation of lipolysis in adipocytes, fatty acid metabolism and phospholipase D signaling pathway (Figure 3). These results suggested that the differentially expressed genes in subcutaneous adipose tissues in LY and LW pigs might be associated with lipid metabolism and adipocyte differentiation. PPAR signaling pathway was the key pathway regulating adipocyte differentiation in LY and LW pigs. Thus, the genes enriched in PPAR signaling pathway might regulate subcutaneous fat deposition and adipocyte differentiation in LW and LY pigs.

### PPI network for differentially expressed genes

**Figure 4:**
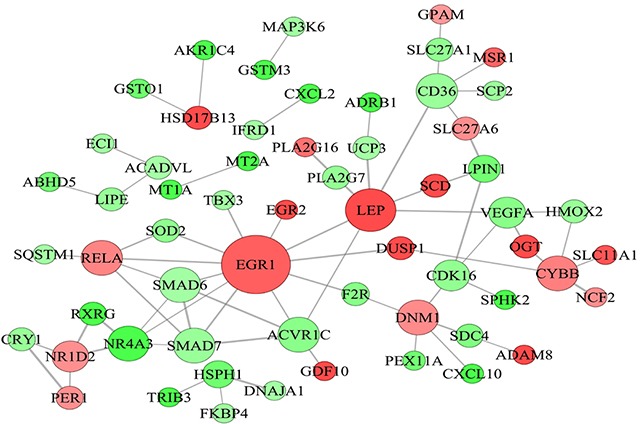
Protein-protein interaction network of differentially expressed genes related to lipid metabolism Node represents protein, edge represents interaction between proteins, size of the node is proportional to degree of this node (degree of the node is defined as amount of proteins that interact with this node), and color of node represents Log2FoldChange in expression levels of differentially expressed genes between Laiwu and Large White pigs.

PPI network for the differentially expressed genes related to lipid metabolism was constructed. On this basis, the candidate genes regulating fat deposition in LY and LW pigs were identified (Supplementary Table 5). In fact, some researchers previously identified candidate genes related to intramuscular fat deposition in cattle by using PPI network, which provided useful information for our study [24]. Gene-encoded proteins including early growth response (*EGR1)*, leptin (*LEP)*, CD36 molecule (*CD36)*, SMAD6 family member 6 (*SMAD6)*, SMAD6 family member 7 (*SMAD7)*, nuclear receptor subfamily 4 group 3 (*NR4A3)*, NADPH oxidase heavy chain subunit (*CYBB*), nuclear receptor subfamily 1 group D member 2 (*NR1D2*), dynamin 1 (*DNM1*), activin receptor 1C (*ACVR1C*) were at hub positions in the PPI network (Figure 4). Thus, they might be important for regulating subcutaneous fat metabolism and adipogenic differentiation in pigs.

### Target genes of lncRNAs and functional analysis

**Figure 5:**
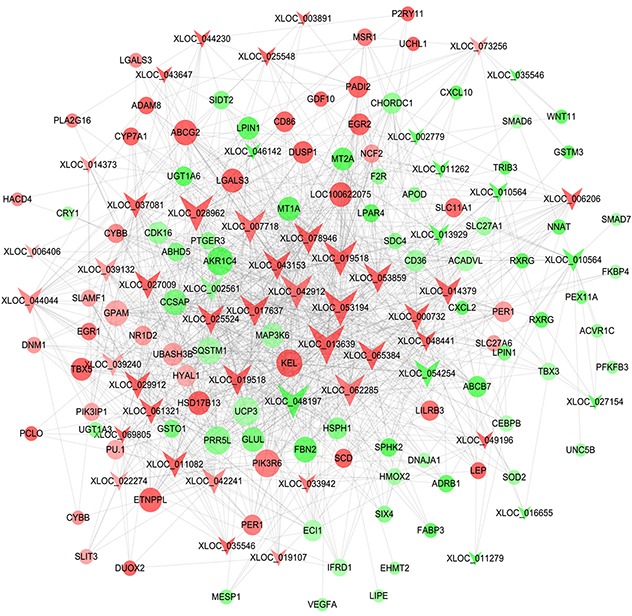
Co-expression network of the differentially expressed lncRNAs and genes related to lipid metabolism Note that color denotes differential expression levels. Red represents up-regulation and green represents down-regulation. Size represents the importance of a node (Degree). The edge denotes the interaction strength. Circles and inverted triangles represent genes and lncRNAs, respectively.

By correlation analysis of the expression levels of lncRNAs and mRNAs, 425 differentially expressed mRNA were found to co-express with differentially expressed lncRNAs (Supplementary Table 6). According to the results of GO and KEGG analyses, the differentially expressed genes related to lipid metabolism, fat-associated diseases and adipocyte differentiation were investigated to identify key lncRNAs regulating fat deposition. LncRNA-mRNA co-expression network with the threshold |PCC|≥0.9 was constructed (Supplementary Table 7). As showed in Figure 5, some lncRNAs were at the central positions of the network. Using Degree (Degree Centrality) ≥ 30 as the threshold, 14 key lncRNAs were selected. They were *XLOC_013639, XLOC_053194*, *XLOC_028962*, *XLOC_019518*, *XLOC_042912*, *XLOC_ 048197*, *XLOC_007718*, *XLOC_062285*, *XLOC_017637*, *XLOC_078946*, *XLOC_053859*, *XLOC_065384*, *XLOC_ 043153* and *XLOC_025524*. These could serve as a basis for further research on lncRNA function.

There were only 5 lncRNAs that were predicted to cis-regulate target genes (Supplementary Table 8). There were many target genes that were trans-regulated by lncRNAs (Supplementary Table 9). Considering genes related to lipid metabolism, four pairs of lncRNA-mRNA were selected (Table 2). By trans-target gene analysis, three key lncRNAs were identified. Combined with the 14 key lncRNAs obtained by co-expression analysis, there were a total of 17 key lncRNAs.

**Table 2:** Trans-target genes related to lipid metabolism of differentially expressed lncRNAs

lncRNA id	lncRNA expression	gene id	mRNA expression
*XLOC_014379*	up	*HSD17B13*	up
*XLOC_014379*	up	*SCD*	up
*XLOC_011279*	down	*LPIN1*	down
*XLOC_064871*	down	*TRIB3*	down

**Figure 6:**
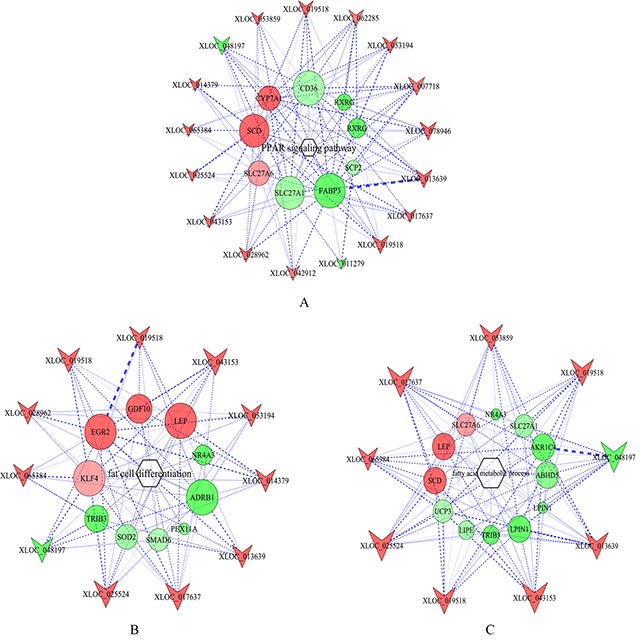
LncRNA-gene-pathway network related to lipid metabolism **(A)** lncRNA-gene-PPAR signaling pathway network. **(B)** lncRNA-gene-fat cell differentiation network. **(C)** lncRNA-gene-fatty acid metabolism process network. Hexagons, circles and inverted triangles represent pathways, genes and lncRNAs, respectively. Note that color denotes differential expression levels. Red represents up-regulation, and green represents down-regulation. Size represents the importance of a node (Degree). The edge denotes the interaction strength.

The target genes of lncRNAs were also enriched in PPAR signaling pathway and biological processes including adipogenic differentiation, fatty acid metabolism and lipid metabolism. This indicated that the differentially expressed lncRNAs might regulate adipogenic differentiation and lipid metabolism by targeting key genes involved in above signaling pathway and biological processes. To further explore the function of key lncRNAs, GO annotation and KEGG enrichment analysis were performed for the target genes of each lncRNA. LncRNAs whose target genes were significantly enriched in PPAR signaling pathways, fat cell differentiation and fatty acid metabolic process were selected for the construction of lncRNA-mRNA-signaling pathway network (Figure 6). According to the network, the regulatory patterns of key lncRNA-mRNA were explored.

### qRT-PCR verification

**Figure 7:**
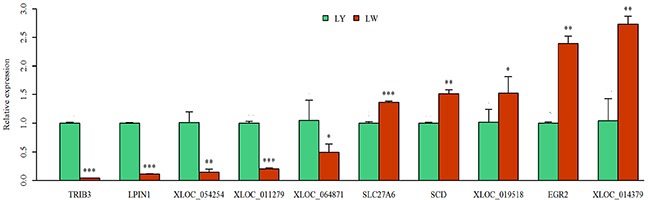
qRT-PCR verification of the differentially expressed genes The differential expression of genes in subcutaneous adipose tissues between Laiwu and Large White pigs was verified by qRT-PCR. ^*^: *P*<0.05; ^**^: *P*<0.01; ^***^: *P*<0.001.

qRT-PCR is considered as the golden standard for quantitative analysis of genes [25]. RNA-Seq data are significantly correlated to qRT-PCR results (R^2^ = 0.97) [26]. Thus, ten differentially expressed genes were randomly chosen for the verification of RNA-seq results (Supplementary Table 10). As shown in Figure 7, tribbles pseudokinase 3 (*TRIB3*), *LPIN1, XLOC_054254, XLOC_011279* and *XLOC_064871* were up-regulated in subcutaneous adipose tissue in LY pig. Solute carrier family 27 member 6 (*SLC27A6*), *SCD*, *XLOC_019518*, early growth response 2 (*EGR2*) and *XLOC_014379* were up-regulated in subcutaneous adipose tissue in LW pig. These results were consistent with the sequencing results, implying the reliability of sequencing results.

## DISCUSSION

Fat content in animals is determined by preadipocyte differentiation, adipocyte differentiation and proliferation, and fat synthesis. Fat deposition is closely related to the genetic background, developmental stage and nutritional level of animals and is regulated by many transcription factors, key genes and signaling pathways. In this study, RNA-seq technology was used to analyze and compare the gene expression profiles of subcutaneous adipose tissues between LW and LY pigs. GO and KEGG enrichment analysis indicated that the differentially expressed genes were mainly involved in PPAR signaling pathway and biological processes including fatty acid metabolism and adipocyte differentiation. Analysis of differential lncRNA-mRNA co-expression patterns and functional analysis of target genes indicated that lncRNAs regulated subcutaneous fat metabolism and deposition by regulating genes associated with above-mentioned signaling pathway and biological processes. In sum, this study explored the role of lncRNAs in regulating subcutaneous fat deposition in pig and might provide a basis for studying the molecular mechanism of lncRNAs regulating adipogenic differentiation and lipid metabolism.

PPAR signaling pathway is a key pathway closely related to fatty acid and sterol metabolism as well as adipogenic differentiation [27]. In this study, PPAR signaling pathway was significantly enriched with eight differentially expressed genes: *CD36*, cytochrome P450 family 7 subfamily A polypeptide 1 (*CYP7A1*), fatty acid binding protein 3 (*FABP3*), *RXRG*, *SCD*, sterol carrier protein 2 (*SCP2*), solute carrier family 27 member 1 (*SLC27A1*) and *SLC27A6*. These 8 differentially expressed genes were regulated by 17 lncRNAs. *CD36* that positively correlates with the expression of *PPARγ* can facilitate cellular uptake of LCFA and promote adipocyte differentiation and fat biosynthesis [28]. *CYP7A1* is a key rate-limiting enzyme for transformation of cholesterol to bile acid, and its activity is negatively correlated with the levels of plasma low-density lipoprotein and cholesterol in rodents and humans [29, 30]. *SCP2*, by regulating protein and lipid compositions in lipid droplets [31], can inhibit HDL-mediated efflux of sterol from lipid droplets [32]. The lipid deposition in mice with the absence of *SCP2* is significantly increased [33], indicating that *SCP2* is conducive to lipid maintenance. *SLC27A1* and *SLC27A6* are genes encoding proteins belonging to fatty acid transport protein (SLC27 protein) family. SLC27 proteins are key regulator of fatty acid metabolism and can help maintain lipid homeostasis and promote uptake of LCFA and lipid deposition [34].

*SCD* is a key enzyme transforming saturated fatty acid to endogenous oleic acid in food and regulating unsaturated fatty acid biosynthesis, and it can promote lipid deposition [35]. In this study, *SCD* was up-regulated in subcutaneous adipose tissue of LW pig, which was consistent with previous study showing that *SCD* was highly expressed in subcutaneous adipose tissue of pigs with thick backfat [8, 36]. The backfat thickness of LW was higher than that of LY, implying that *SCD* promoted subcutaneous fat deposition in LW. Meanwhile, *XLOC_014379* might target *SCD* and involve in PPAR signaling pathway, thus regulating fatty acid metabolism. Previous study indicated that the expression level of *FABP3* in subcutaneous adipose tissue was positively correlated with the backfat thickness in cattle [37]. In this study, however, *FABP3* was down-regulated in subcutaneous adipose tissue in LW, implying that the mechanisms of *FABP3* regulating fat deposition in pig and cattle might be different. The above results indicated that the genes enriched in PPAR signaling pathway were mainly involved in fatty acid and sterol metabolism, which could influence lipid deposition. LncRNAs such as *XLOC_013639* (Figure 6) might regulate PPAR signaling pathway by regulating these genes, thus playing an important role in fatty acid metabolism and lipid deposition in subcutaneous adipose tissue in LW and LY pigs. Especially, *XLOC_014379* might target *SCD* and play a crucial role.

GO annotation demonstrated that differentially expressed genes were mainly associated with fatty acid metabolism and adipocyte differentiation. There were 9 and 11 key lncRNAs in fatty acid metabolism network and adipocyte differentiation network, respectively. Thus, the function of these lncRNAs should be closely related to fatty acid metabolism and adipogenic differentiation. After excluding the genes associated with PPAR signaling pathway, 14 genes including abhydrolase domain containing 5 (*ABHD5*), *AKR1C4*, lipase E (*LIPE*), *LPIN1*, *NR4A3*, *TRIB3*, uncoupling protein 3 (*UCP3*), peroxisomal biogenesis factor 11 alpha (*PEX11A*), *SMAD6* and superoxide dismutase 2 (*SOD2*) were up-regulated in subcutaneous adipose tissue in LY pig, whereas *LEP*, *EGR2*, growth differentiation factor 10 (*GDF10*) and *KLF4* were up-regulated in subcutaneous adipose tissue in LW pig. *In vitro* study indicates that *ABHD5* can activate adipose triglyceride lipase (ATGL) and promote lipid decomposition [38]. *LPIN1* is important for adipocyte differentiation and lipid metabolism [39] and is a candidate gene regulating fat deposition in pig [7]. Some research demonstrates that *LPIN1* mRNA level is positively correlated with insulin sensitivity in subcutaneous adipose tissue of human [40]. In this study, *XLOC_011279* might involve in subcutaneous adipogenesis, lipid metabolism and insulin sensitivity by targeting *LPIN1*. NR4A3 belongs to nuclear hormone receptor (NR) family that can regulate lipid and carbohydrate metabolism [41]. NR4A3, a target of beta-adrenergic signaling, play a key role in regulating fatty acid oxidation in skeletal muscle in mice, and muscle-specific *NR4A3* expression can reduce deposition of white and brown fat [42, 43]. Another research suggests *NR4A3* could promote the differentiation of myocytes into myotubes in biceps of pig [44]. In this study, *NR4A3* was up-regulated in subcutaneous adipose tissue in LY pig, indicating it might inhibit adipogenic differentiation. However, PPI network demonstrated that *NR1D2*, another member in the NR family, was highly expressed in subcutaneous adipose tissue of LW pig. *NR1D2* can induce the expression of *PPARs*, *CEBPα* and *FABP4* genes and promote adipogenic differentiation in dwarf chickens [45]. Thus, *NR1D2* might promote adipogenic differentiation in subcutaneous adipose tissue of LW pig. TRIB3, a kind of pseudokinase, can inhibit synthesis of fatty acid by combining with E3 ubiquitin ligase and protect against diet-induced obesity by stimulating fatty acid oxidation in adipose of mice during fasting [46]. In addition, *TRIB3* can negatively regulate the transcriptional activation of PPARγ and inhibit adipogenesis in 3T3-L1 cells [47]. *TRIB3* is expressed in porcine adipose tissue and skeletal muscle, and is related to meat quality and production performance [48]. These indicate that *TRIB3* might inhibit subcutaneous fat deposition in LY pig and be closely related to obesity-associated diseases. In this study, *XLOC_064871* can trans-regulate *TRIB3*, indicating that *XLOC_064871* might also play a vital role in adipocyte differentiation and fatty acid metabolism in pig. *UCP3* is a negative regulator of energy balance and lipid metabolism. By inhibiting triglycerides storage in cytoplasm, *UCP3* gene can affect lipid metabolism and inhibit fat deposition [49, 50]. Polymorphism analysis indicates that *UCP3* gene can help determine backfat thickness and intramuscular fat content of pig [51] and cattle [52]. Thus, the high expression level of *UCP3* gene might negatively regulate subcutaneous fat deposition in LY pig. *SMAD6* and *SMAD7* are mainly associated with TGFβ signaling pathway. In 3T3-F442A preadipocytes, over-expression of *SMAD6/7* can inhibit early differentiation of adipocytes and thus negatively regulate adipogenesis [53]. Accordingly, the high expression level of *SMAD6/7* might inhibit pig fat deposition. On above basis, all these genes that were highly expressed in subcutaneous adipose tissue of LY pig might negatively regulate adipocyte differentiation and lipogenesis. In other words, these genes may inhibit subcutaneous fat deposition in LY pig, leading to lower backfat thickness than that of LW pig.

Compared with genes in subcutaneous adipose tissue in LY pig, those in LW pig were highly expressed, mainly promoting adipogenesis in LW pig. EGR2 and KLF4 are important zinc finger transcription factors promoting early adipocyte differentiation, and can stimulate adipogenesis by trans-activating *C/EBPβ* in 3T3-L1 preadipocytes [54, 55]. In LY pigs with different backfat thicknesses, *EGR2* is highly expressed in pig with thicker backfat [36], which is consistent with our results. Taken together, subcutaneous adipocytes in LW pig might have greater differentiation ability than those in LY pig, leading to significant difference in subcutaneous fat deposition between the two pig breeds. This was consistent with the results of Zhang et al [56]. In sum, key lncRNAs played an important role in regulating subcutaneous adipocyte differentiation and affecting fat deposition in LW and LY pigs. *XLOC_011279* and *XLOC_064871* can target *LPIN1* and *TRIB3*, respectively, and might play a crucial role in adipogenic differentiation. In addition, they could be potential targets for therapy of obesity-associated diseases.

## MATERIALS AND METHODS

### Ethic statement

All the procedures involving animals were approved by the Animal Care and Use Committee at Institute of Animal Sciences, Chinese Academy of Agricultural Sciences where the experiments were conducted. All the experiments were performed in accordance with relevant guidelines and regulations set by the Ministry of Agriculture of the People's Republic of China.

### Animal and sample preparation

In this study, three healthy female Large White pigs (LYs) and three healthy female Laiwu pigs (LWs) fed a diet formulated to meet current nutritional requirements were selected and raised at Daqian Farming Co. Ltd. of Laiwu (Laiwu city, Shangdong Province, China). All animals had free access to water and food under natural lighting. At slaughter age (180 days old), the mean bodyweight of pigs was 108 ± 2 kg. After slaughter, the subcutaneous adipose tissue of longissimus dorsi muscle was sampled into 5 mL tubes within 30 min, frozen in liquid nitrogen immediately and then transferred to refrigerator at −80° for long-term preservation and further total RNA extraction.

### Isolation of total RNA and quality control

The same amount of adipose tissue samples were taken for total RNA isolation. mirVana^TM^ RNA Isolation Kits (#AM1561, Ambion, USA) were used to isolate total RNA according to the manufacturer's instruction. The isolated total RNA from each sample was preserved at −80°. NanoDrop 2000 spectrophotometer (Thermo Scientific, USA) was used to determine RNA concentration and *OD*_260 nm_/*OD*_280 nm_ absorption ratio, which was controlled in the range of 1.9–2.1. Bioanalyzer 2100 (Agilent, Santa Clara, CA) was used to evaluate the quality of total RNA (RIN>=7 and 28S/18S>=0.7). RNase-free DNase I (Ambion Inc., Texas, USA) was used to eliminate potential genomic DNA contamination.

### cDNA library construction and RNA sequencing

Approximately 1 μg of total RNA from each sample was used for the construction of cDNA libraries (including LW1, LW2 and LW3; LY1, LY2 and LY3) according to Illumina® TruSeq™ RNA Sample Preparation Guide. The process includes (1) separation, enrichment and purification of mRNA using oligo (dT) magnetic beads, (2) enzymatic fragmentation of RNA, (3) synthesis of cDNA, (4) sequencing adapter ligation and (5) PCR amplification. Agilent DNA 1000 Kits on Agilent 2100 Bioanalyzer (Agilent technologies, Santa Clara, CA) was used to determine the size and purity of cDNA libraries. ABI StepOnePlus Real-time fluorescence-based quantitative PCR system was used to accurately determine the effective concentration of cDNA libraries (> 2 nmol/L), thus ensuring the quality of cDNA library. Illumina HiSeq^TM^2500 platform was used for paired-end sequencing of cDNA libraries and raw reads were obtained.

### Reference genome mapping and transcriptome assembly

Clean reads were obtained by removing reads containing adapter contamination, reads containing more than 10% poly-N, and low-quality reads (containing more than 15% bases with *Q*-scores no higher than 20) using fastx_toolkit software (v0.0.14). TopHat [57, 58] (v2.0.12) was used to align clean reads of each sample to reference genome Sscrofa10.2 (ftp://ftp.ensembl.org/pub/release-87/fasta/sus_scrofa/dna/). Reads were located and mismatch was set to 2 (all other parameters were set to their default values). Cufflinks [58] (v2.1.1) was used for transcriptome assembly and reconstruction. Then, the transcripts were aligned to reference annotation files Sscrofa10.2.87.chr.gtf (ftp://ftp.ensembl.org/pub/release-87/gtf/sus_scrofa). The known lncRNA and mRNA transcripts were thus identified. Meanwhile, the position of transcripts was obtained.

### Identification of potential lncRNA candidates

LncRNAs are non-protein coding transcripts longer than 200 nucleotides. Potential lncRNA candidates were identified according to these two characteristics. The detailed process was as follows. (1) Identification based on transcript type. The transcripts whose class code were ‘i’, ‘u’, ‘x’ and ‘o’ were screened for identification of potential lncRNA candidates, which corresponded to intronic lncRNA, lincRNA, antisense lncRNA and sense lncRNA, respectively. (2) Identification based on exon number and transcript length. Exon number ≥2 and transcript length > 200 bp were taken as the threshold. Single-exon transcripts with low credibility were filtered out. (3) Identification based on the protein-coding potential of transcripts. PLEK [59], CNCI [60], CPC [61] and Pfam [62] were applied to assess the protein-coding potential of the above-obtained transcripts and the intersection of results from these software was as the finally result. (4) Identification of known lncRNAs. ALDB (A Domestic-Animal Long Noncoding RNA Database) [63] is a database with a focus on the domestic-animal lncRNAs. BLASTN tool was used to align lncRNA candidates to lncRNAs in the database with the setting of identity=100%, mismatch=0, E-value<1e-10 and gap_opening=0. Then, the already known lncRNAs were identified.

### Analysis of differentially expressed genes

The expression levels of lncRNAs and mRNAs were normalized using fragments per kilobase of transcript per million mapped reads (FPKM) algorithm, eliminating the influence of sequencing depth, gene length and sample difference on gene expression level. For the experiment with three biological replicates, identification of differentially expressed lncRNAs and protein-coding genes was performed using R package DESeq2 [64] following negative binomial distribution. Multiple hypothesis testing correction of P value was performed with Benjamini-Hochberg algorithm. If |log2FoldChange| ≥ 1 (LW *vs* LY) and padj (adjusted P value) ≤ 0.05, the genes and lncRNAs were considered to be differentially expressed.

### GO and KEGG pathway enrichment analysis of differentially expressed genes

GO (http://www.geneontology.org/) was the international standard classification of gene function. It classifies functions along three aspects including molecular function, biological process and cellular component. Pathway enrichment analysis can help determine the main metabolic pathways and signaling pathways in which differentially expressed genes are involved. KEGG (http://www.genome.jp/kegg) database [65] is a main public database for metabolic analysis and regulatory network research. To further explore the main biological function of differentially expressed genes, CluGO [66] plugin of Cytoscape, based on hypergeometric distribution, was used to determine the GO terms and signaling pathways that differentially expressed genes were enriched in. Benjamini-Hochberg algorithm was used for P value correction. If Q value≤ (corrected P value) ≤ 0.05, the enrichment was significant.

### Protein-protein interaction (PPI) network analysis for differentially expressed genes

According to STRING database (http://string-db.org/), PPI network analysis was performed for the differentially expressed genes, and the interaction among differentially expressed genes in subcutaneous adipose tissue of LW and LY were further investigated. Cytoscape was used to visualize the obtained PPI network data files, and the key genes were identified.

### Prediction and functional analysis of the target genes of differentially expressed lncRNAs

The main role of lncRNAs, as a kind of non-coding RNAs, is to regulate their target genes: cis-regulating nearby protein-coding genes and trans-regulating distant protein-coding genes. If genes have same expression pattern, their functions will be highly correlated. Therefore, the target genes of lncRNAs were analyzed and key lncRNAs were identified as follows. The Pearson correlation coefficients (PCCs) of expression levels of differentially expressed lncRNAs and mRNAs were calculated. |PCC|>0.8 and P value<0.05 were the threshold according to which co-expressed lncRNA-mRNA were selected. The protein-coding gene located 300 kb upstream and downstream of lncRNA in genome was selected. If |PCC|≥0.9, the gene would be the target gene that was cis-regulated by lncRNA. According to the interaction between lncRNA and mRNA sequences, the target genes that were trans-regulated by lncRNA were predicted. RNAplex [67] was used to calculate the free energy of combination of lncRNA and mRNA sequences. If Energy<-20 and |PCC| ≥ 0.9, the gene would be the target gene that was trans-regulated by lncRNA [68, 69].

GO annotation and KEGG pathway enrichment analysis were performed for the obtained target genes of lncRNAs. Thus, the biological processes and signaling pathways that lncRNAs were enriched in were identified. Then, the functions of lncRNAs were predicted.

### Real-time fluorescence-based quantitative PCR (qRT-PCR) verification

qRT-PCR was applied to verify the expression level of genes. Ten differentially expressed genes (five lncRNAs and five mRNAs) were randomly selected. Three biological replicates were employed for each gene. GeneAmp® PCR System 9700 (Applied Biosystems, USA) was used to synthesize cDNA templates via reverse transcription. Approximately 0.5 μg of each RNA sample was used. QuantiFast^®^ SYBR^®^ Green PCR Kit (Qiagen, Germany) and LightCycler^®^ 480 II Real-time PCR Instrument (Roche, Swiss) were used for qRT-PCR analysis. The reaction system consisted of 1 μL of cDNA, 5 μL of 2× QuantiFast^®^ SYBR^®^ Green PCR Premix (Qiagen, Germany), 0.2 μL of forward primer, 0.2 μL of reverse primer and 3.6 μL of nuclease-free water. Reaction conditions were as follows: pre-denaturation at 95 ° for 5 min, followed by 40 cycles; denaturation at 95 ° for 10 s; renaturation at 60 ° and extension for 30 s. Pig β-actin (*ACTB*) gene was used as the internal control and 2^−ΔΔCt^ method was used to calculate the relative expression levels of genes between samples.

### Statistics analysis

All the data were presented as “means ± SDs”. When comparisons were made, a Student's *t*-test was performed and *P* < 0.05 was considered statistically significant.

## CONCLUSIONS

In this study, RNA-seq technology and bioinformatics methods were applied to identify the differentially expressed lncRNAs and genes of subcutaneous adipose tissues between Laiwu and Large White pigs. On this basis, the molecular mechanism of fat deposition was explored. Results indicated that 54 lncRNAs and 482 known genes were differentially expressed. LncRNAs can target mRNAs and then play an important role in PPAR signal transduction pathway, adipocyte differentiation and fatty acid metabolism. By this way, they regulated subcutaneous fat deposition in Laiwu and Large White pigs. *XLOC_014379*, *XLOC_011279*, *XLOC_064871, XLOC_019518 and XLOC_013639*, which might target *SCD*, *LPIN1, TRIB3, EGR2 and FABP3*, respectively, play key regulatory roles. This study can provide useful information for understanding molecular mechanism of fat deposition in pig. In addition, this study may help breed pig with high meat quality as well as prevent and treat disease associated with fat metabolism.

## SUPPLEMENTARY MATERIALS TABLES






















