# Supplementary material for: Differential regulation of mRNAs and lncRNAs related to lipid metabolism in two pig breeds
Source: Oncotarget. 2017 Sep 18;8(50):87539–53. doi: 10.18632/oncotarget.20978 (PMC5675652; doi:10.18632/oncotarget.20978)
Supplement: Supplementary file 1 [file oncotarget-08-87539-s001.pdf]

Differential regulation of mRNAs and lncRNAs related to lipid metabolism in two pig breeds

SUPPLEMENTARY MATERIALS

Supplementary Table 1: Differentially expressed genes in subcutaneous adipose tissue between Laiwu and Large White pig.

See Supplementary File 1

Supplementary Table 2: Differentially expressed lncRNAs in subcutaneous adipose tissue between Laiwu and Large White pig.

See Supplementary File 2

Supplementary Table 3: Gene ontology enrichment analysis of differentially expressed genes.

See Supplementary File 3

Supplementary Table 4: KEGG pathway enrichment analysis of differentially expressed genes.

See Supplementary File 4

Supplementary Table 5: Protein-protein interaction network of differently expressed genes related to lipid metabolism.

See Supplementary File 5

Supplementary Table 6: Co-expression analysis of differentially expressed lncRNAs and mRMAs.

See Supplementary File 6

Supplementary Table 7: Co-expression network construction of differentially expressed lncRNAs and mRNAs related to lipid metabolism.

See Supplementary File 7

Supplementary Table 8: Cis-regulated target genes of differentially expressed lncRNAs.

See Supplementary File 8

Supplementary Table 9:Trans-regulated target genes of differentially expressed lncRNAs.

See Supplementary File 9

Supplementary Table 10: qRT-PCR validation of the differentially expressed genes.

See Supplementary File 10
